# Munchausen Syndrome Presenting with Hematemesis And school refusal: A Rare Case Report

**DOI:** 10.1192/j.eurpsy.2023.2066

**Published:** 2023-07-19

**Authors:** S. Singh

**Affiliations:** psychiatry, neuropsychiatric and medical surgical centre, Pathankot, India

## Abstract

**Introduction:**

Factitious hematemesis is the bleeding type of Munchausen’s syndrome together with dual diagnosis of school refusal is rarely reported in the literature. It is a condition in which the patient intentionally produces symptoms to assume a sick role and gain medical attention. Underdiagnosis of this disorder results in the unnecessary use of medical resources, i.e. unnecessary medical tests and evaluations.

**Objectives:**

case

We present this rare case of a patient with chronic factitious disorder who presented to the emergency with hematemesis. The 12 year old male patient grade 6 student presented with curious history of hematemesis just before the entrance of school and in the new school premises since 2 years resulting in school refusal and multiple doctor shopping. The patient underwent laboratory tests (such as the examination of sputum specimens, urinalysis, complete blood evaluations) and diagnostic studies (fiberoptic bronchoscopy with bronchoalveolar lavage, computerized tomography and radiography of the chest, bronchial arteriography, endocopic studies etc), because he continually presented with hematemesis, in order to spot and discover the nature of the bleeding. Since such examinations failed (a few of them-namely fiberoptic bronchoscopies--were even performed when he was coughing up blood) and psychiatric consultations revealed the presence of psychologically traumatic events in the patient’s history which could explain the psychopathic traits of her personality (in fact she was aggressive and unstable in interpersonal relations), a diagnosis of factitious hematemesis in Munchausen’s syndrome was made.

**Methods:**

The typical characteristics that should prompt the physician to include Munchausen syndrome in the diagnosis include deliberately lying, repeatedly coming to the clinic/hospital with similar complaints in a short span of time, taking excessive drugs (especially insulin and warfarin) to induce side-effects, recurrent abdominal pain, scars on limbs, and rheumatologic and hematological disorders.

**Results:**

We recommend that physicians all across the globe should report more cases of Munchausen syndrome. More research is required in this arena to understand the cultural, social, and psychological aspects of Munchausen syndrome and to find out which treatment strategy can be most beneficial for such patients

**Conclusions:**

Munchausen syndrome is a diagnostic dilemma that needs to be given adequate medical and social attention by encouraging further research and spreading awareness not only amongst the general population but also health care providers. With proper evaluation, diagnosis, and psychotherapy, the disease will not remain a diagnostic dilemma and would be easier to control and treat. This case report will contribute towards the awareness of physicians about Munchausen syndrome and the strategies to diagnose and treat it.

**Disclosure of Interest:**

None Declared

